# Supplementary material for: Bisphenol A Diglycidyl Ether-Primary Amine Cooligomer-poly(ε-caprolactone) Networks: Synthesis and Characterization
Source: Polymers (Basel). 2023 Jul 3;15(13):2937. doi: 10.3390/polym15132937 (PMC10346554; doi:10.3390/polym15132937)
Supplement: Supplementary file 1 [file polymers-15-02937-s001.zip › polymers-2459875-supplementary.pdf]

polymers

Supplementary Material: Bisphenol A Diglycidyl Ether-Primary Amine Cooligomer-poly(ε-caprolactone) Networks: Synthesis and Characterization

Katalin Czifrák, Csilla Lakatos, Gabriella Szabó, Bence Vadkerti, Lajos Daróczi, Miklós Zsuga and Sándor Kéki

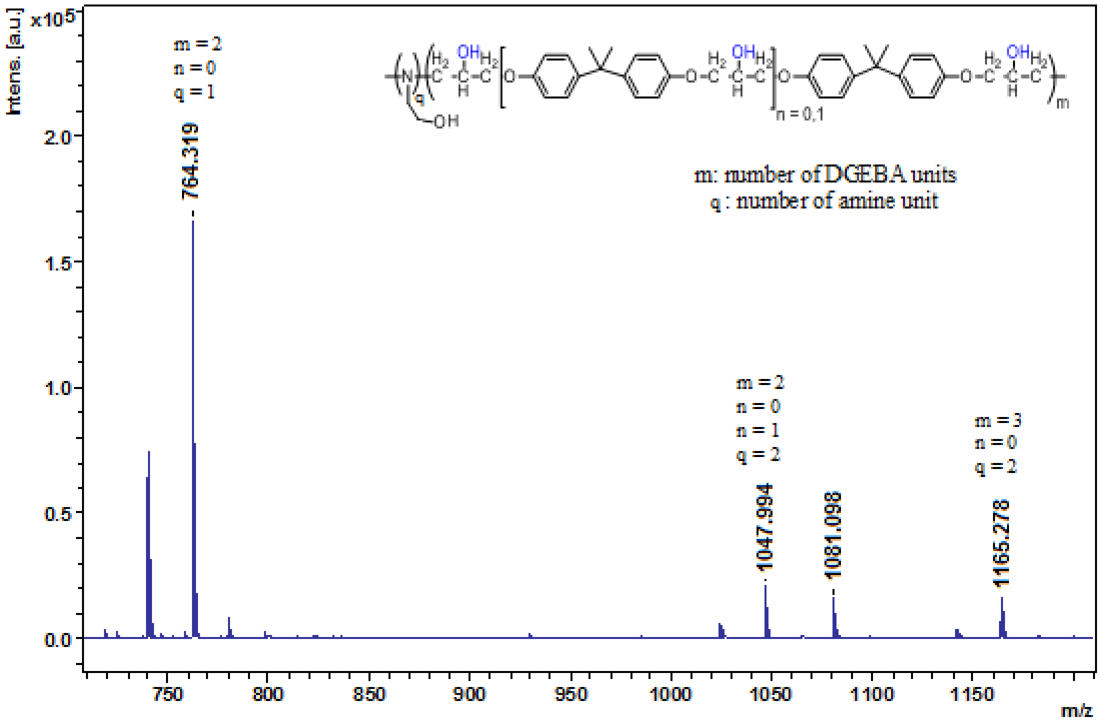

Figure S1. MALDI-TOF MS spectrum of DGEBA-EA / 1:0.2 cooligomer after 2 hrs.

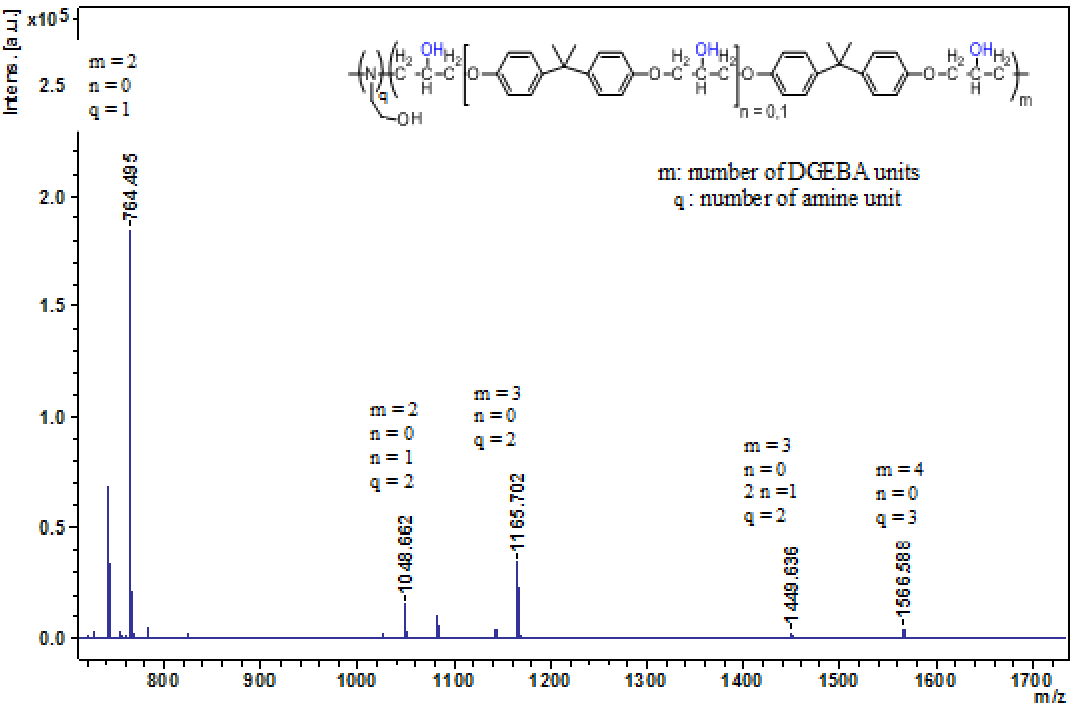

Figure S2. MALDI-TOF MS spectrum of DGEBA-EA / 1:0.3 cooligomer after 2 hrs.

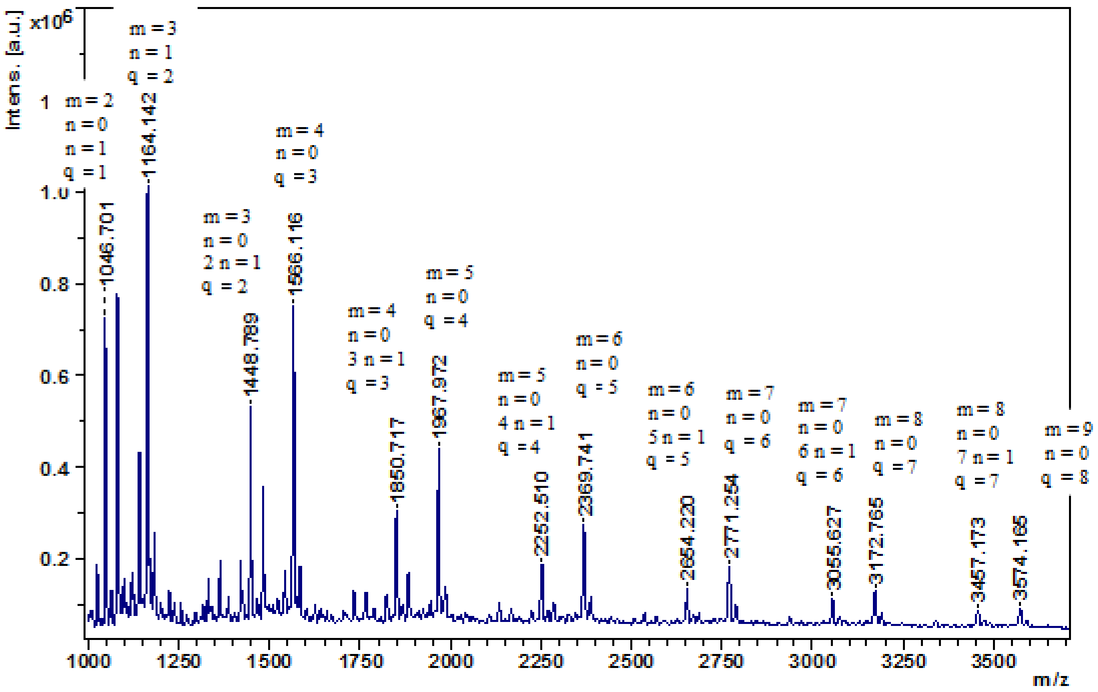

Figure S3. MALDI-TOF MS spectrum of DGEBA-EA / 1:0.3 cooligomer after 2 hrs in linear mode.

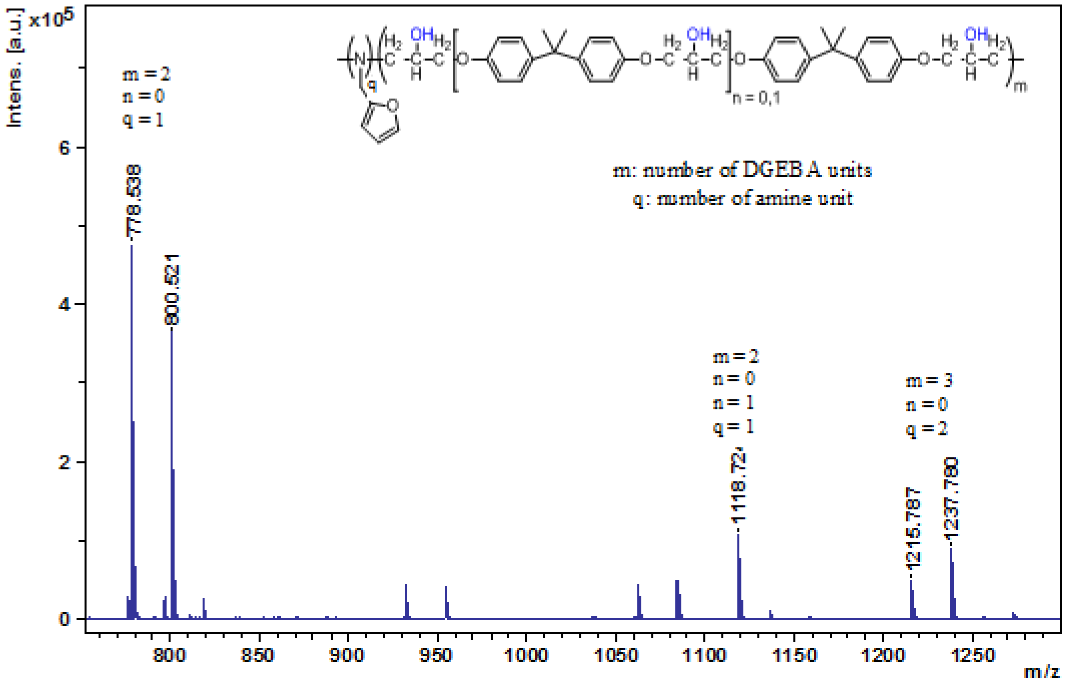

Figure S4. MALDI-TOF MS spectrum of DGEBA-FA / 1:0.1 cooligomer after 2 hrs.

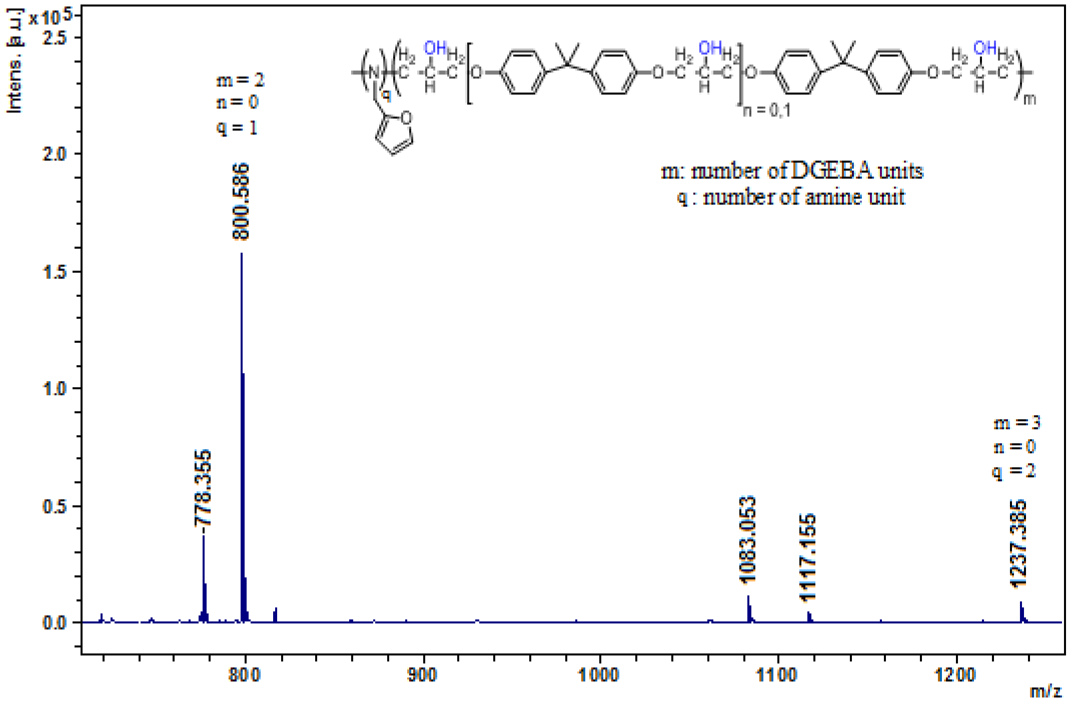

Figure S5. MALDI-TOF MS spectrum of DGEBA-FA / 1:0.2 cooligomer after 2 hrs.

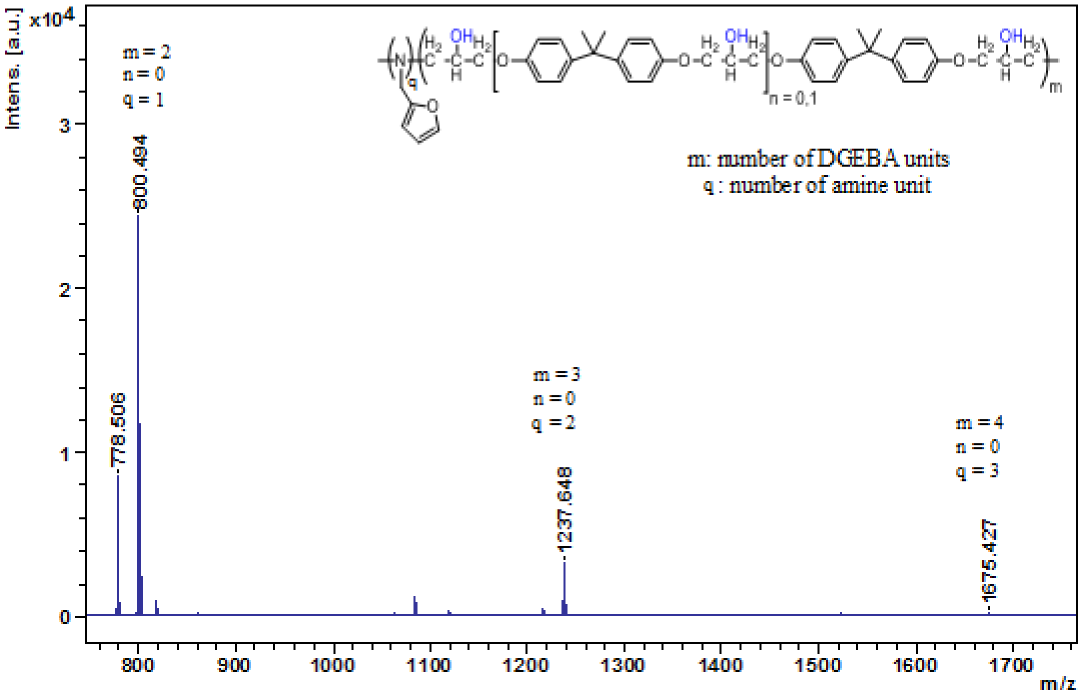

Figure S6. MALDI-TOF MS spectrum of DGEBA-FA / 1:0.3 cooligomer after 2 hrs.

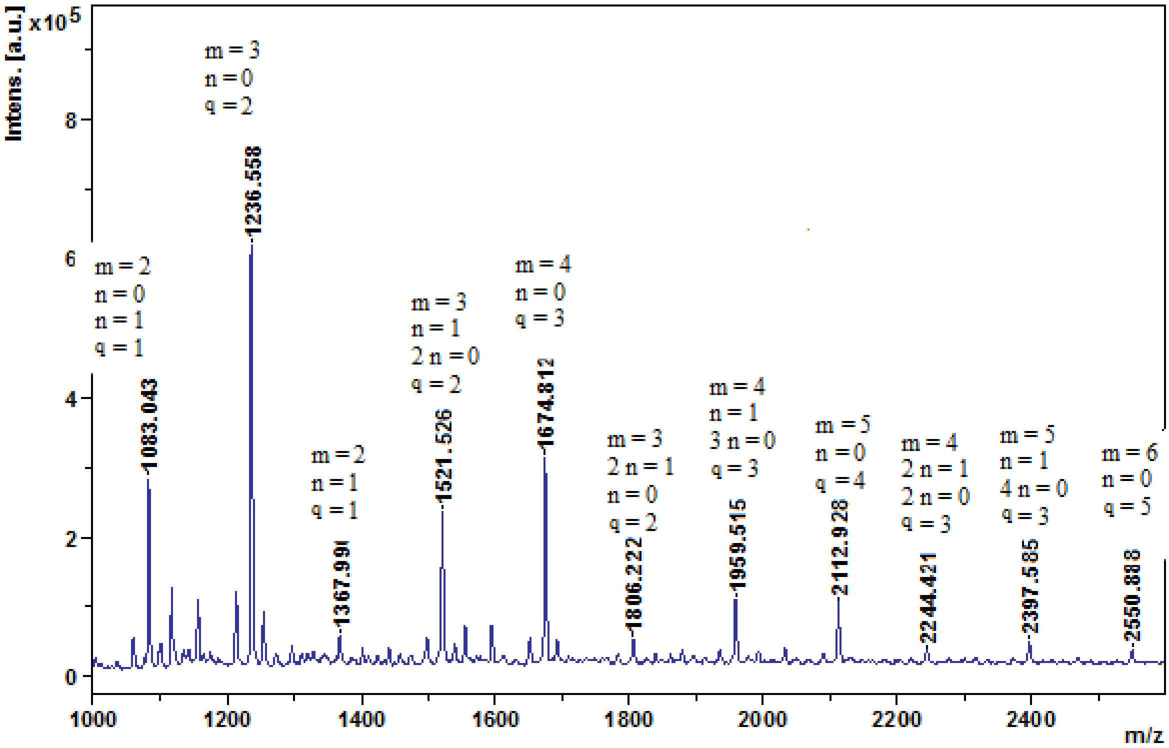

Figure S7. MALDI-TOF MS spectrum of DGEBA-FA / 1:0.3 cooligomer after 2 hrs in linear mode.

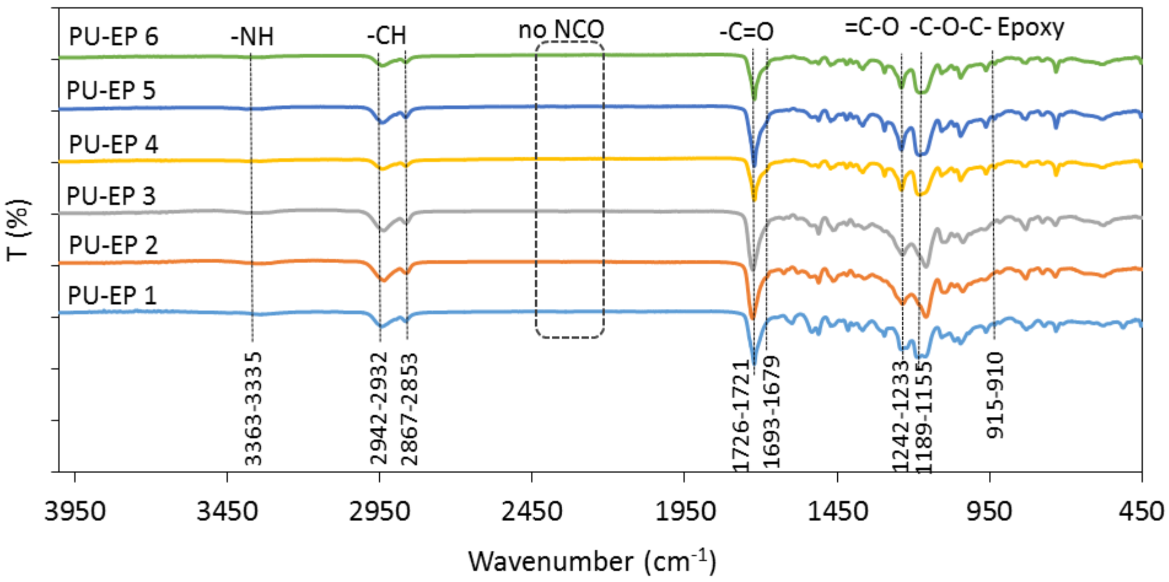

Figure S8. The IR spectra of PU-EPs 1-6.

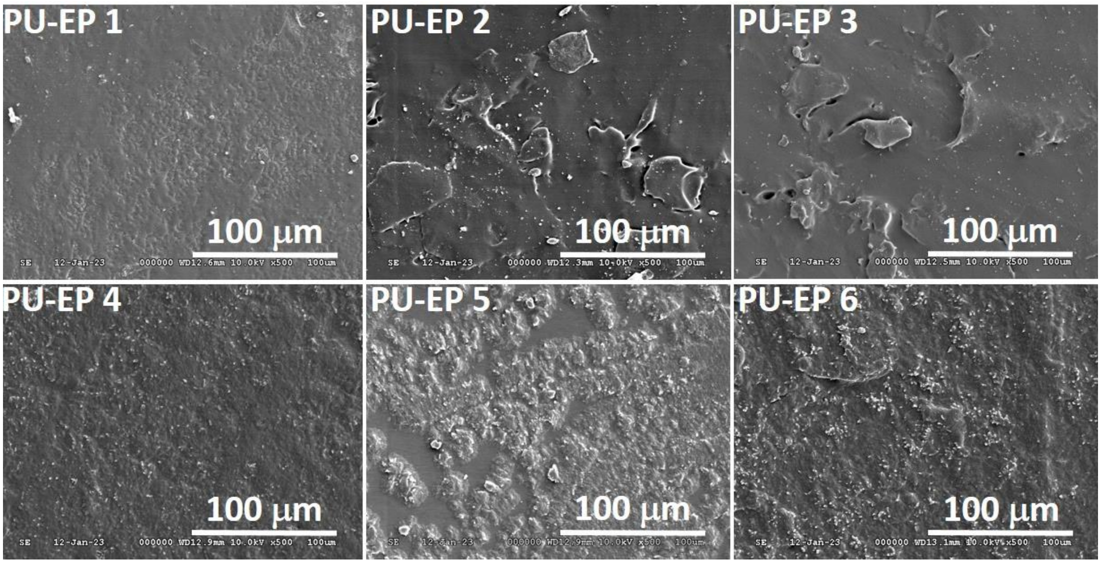

Figure S9. SEM images of samples PU-EPs 1-6.

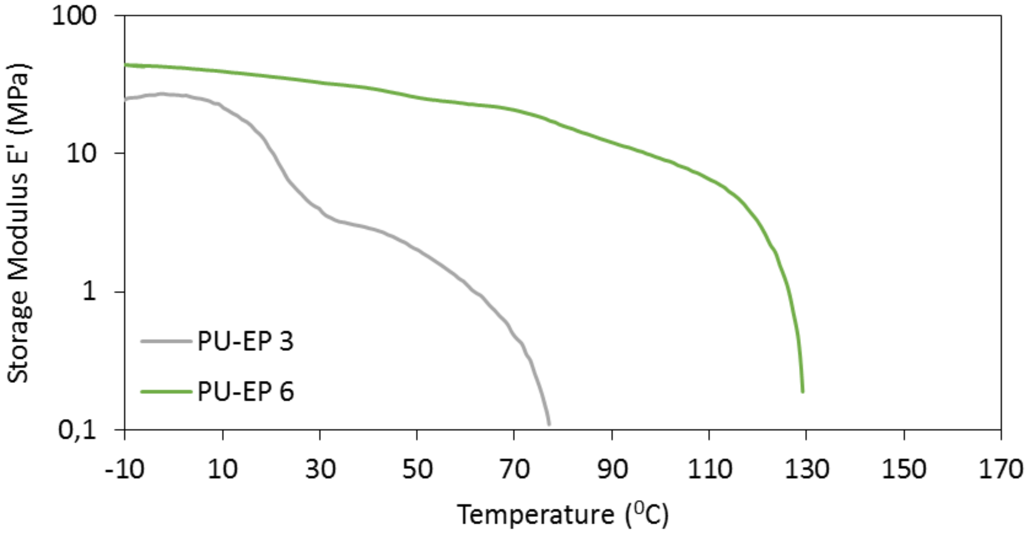

Figure S10. Storage modulus curves of PU-EPs 3 and 6.
